# Activating Photoreaction of Indazole Metastable-State Photoacid through Two-Photon Excitation

**DOI:** 10.1021/acs.jpclett.6c02370

**Published:** 2026-09-12

**Authors:** Ying-Zhong Ma, Pavithra Liyanage, Yi Liao

**Affiliations:** † Chemical Sciences Division, 6146 Oak Ridge National Laboratory, Oak Ridge, Tennessee 37831, United States; ‡ Department of Chemistry and Chemical Engineering, 5401 Florida Institute of Technology, Melbourne, Florida 32901, United States

## Abstract

The photoreaction and functionalities of metastable state photoacids (mPAHs) have been examined almost exclusively through one-photon excitation so far. Here, we report two-photon excited fluorescence measurements on an aqueous solution of a highly water-soluble indazole mPAH. The readily observable fluorescence emission was found to not only follow the anticipated quadratic intensity dependence but also possess a sizeable two-photon absorption cross-section of ∼53 GM. The distinct time-resolved fluorescence decays and emission spectra observed upon one- and two-photon excitation offer indirect evidence for the activation of different isomers and their associated pathways in the respective photoreactions. Our findings pave a promising way of unlocking new functionalities and applications of these mPAHs that are inaccessible to one-photon excitation.

## Graphical abstract

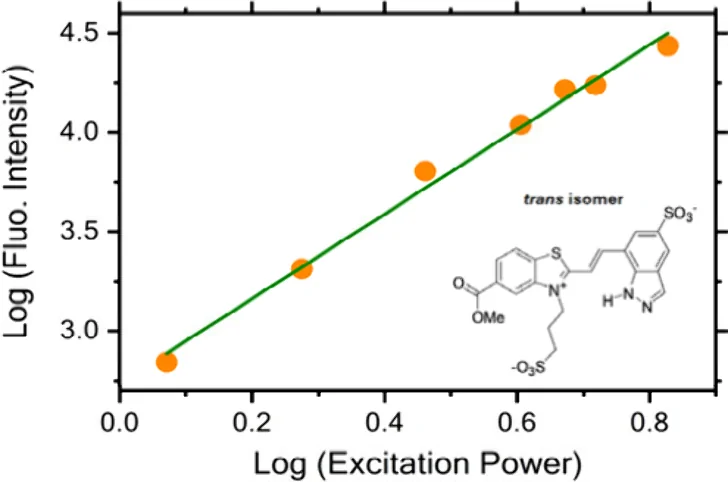

Metastable-state photoacids (mPAHs) are capable of reversibly generating a high proton concentration and a long-lived acidic state during their photoreaction.^1–3^ These unique capabilities have stimulated not only numerous exciting applications^2,4–12^ but also fundamental studies to identify the underlying photoinduced structural changes (PSCs) and competing electronic excited-state processes using a variety of optical spectroscopic techniques. So far, all the available reports have been based on either steady-state or time-resolved optical spectroscopic measurements upon one-photon excitation,^1,3,11,13–19^ except two studies.^20,21^

Two-photon excitation of mPAHs offers direct access to the electronic excited-state(s) that are often inaccessible to one-photon excitation. This can lead to potentially distinct photoreaction pathways, PSCs, and excited-state relaxation processes, which can in turn promote new capabilities and applications. Here, we report a two-photon excited fluorescence (TPEF) spectroscopic study on a highly water-soluble indazole mPAH using time- and frequency-resolved fluorescence (TFRF) spectroscopy. Upon excitation with ∼50 fs pulses centered at 804 nm, we observed a readily detectable fluorescence emission in the similar spectral region as commonly found with one-photon excitation, which is also directly visible to the unaided eye. The fluorescence intensity scales quadratically with excitation intensity, validating its two-photon excitation origin. This validation also enabled us to estimate the two-photon absorption cross-section (σ) for this mPAH using a comparative method. Direct comparison of the time-resolved fluorescence (TRF) decays upon one- and two-photon excitation, as well as the time-integrated TPEF emission spectrum with one-photon excited steady-state emission spectrum further reveals both similarities and differences. The observed differences offer indirect evidence for the involvement of different photoproducts and pathways in the respective photoreactions. This finding suggests a potential route towards unlocking new functionalities and applications of these mPAHs via two-photon excitation.

The water-soluble indazole mPAH employed here differs structurally from other mPAHs with the attachment of a SO_3_® substituent on the indazole moiety and the addition of an ester group to the benzothiazolium side as shown in Figure 1a.^11^ The reaction mechanism of this indazole mPAHs has been the subject of a previous study.^22^ Specifically, it was revealed that photoirradiation of this indazole mPAH generates a quinoidal *cis* isomer in water, which possesses a higher acidity than that of the *trans* isomer due to the unusual quinoidal structure. The deprotonated *cis* isomer was further found to be the intermediate for both the *spiro* formation and the reverse *cis*-to-*trans* reaction. In view of the slightly higher acidity of the *cis* isomer than that of the *trans* isomer, but the acidity of the *spiro* form is significantly greater, it was suggested that the proton transfer occurs either synchronically with or sequentially after the conversion of the *cis* isomer to the *spiro* form. In either case, this proton transfer is known to be preceded by the corresponding *trans*–*cis* photoisomerization, and based on the determined time scale of approximately 25 ps for the *trans*–*cis* photoisomerization of a different indazole mPAH in neat water,^17^ it is reasonable to consider that the proton transfer takes place in a time scale greater than 25 ps for the indazole mPAH examined in this study. Moreover, the reverse reaction rate of this indazole mPAH was measured by acquiring absorption spectra at different times upon switching off irradiation. As shown in Figure S1 in the Supporting Information, the spectra recover to the initial *trans* isomer in 20 min. By fitting the data to a first order reaction equation, which is commonly used for mPAHs,^23^ the reverse reaction rate was calculated to be 1.6 × 10^–3^ s^–1^, corresponding to a half-life of 433 s or 7.2 min.

**Figure 1:**
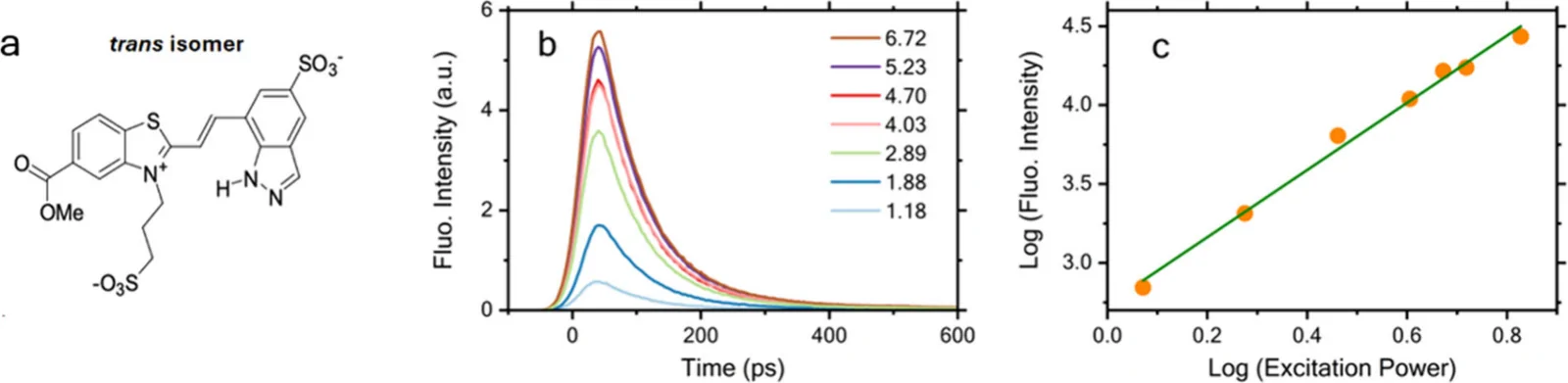
(a) Molecular structure of the *trans* isomer of the indazole mPAH examined in this work. (b) TRF decays measured using a 1 mM aqueous solution of the indazole mPAH under different excitation intensities depicted by the corresponding average powers in mW. (c) Log–log plot of the time-integrated TRF intensities as a function of the average powers of two-photon excitation. The solid line in (c) is the linear fit to the experimental data.

Upon excitation with ∼50 fs pulses centered at 804 nm, we found that the TPEF signal from an aqueous solution can be readily observed. The resulting TPEF is also directly visible to the unaided eye as shown in Figure S2. TRF decays acquired at 560 nm under different excitation intensities of ∼50 fs pulses centered at 804 nm are shown in Figure 1b. A log–log plot of the resulting integrated fluorescence intensity over the entire temporal range of the detectable emission as a function of the excitation power are shown in Figure 2c. A linear fit to this log–log plot gives a slope of 2.13 ± 0.09 and an intercept of 2.74 ± 0.05. This slope confirms the anticipated quadratic intensity dependence for the obtained TPEF signals. This result, to the best of our knowledge, represents the first observation of TPEF emission from all currently available mPAHs.

**Figure 2:**
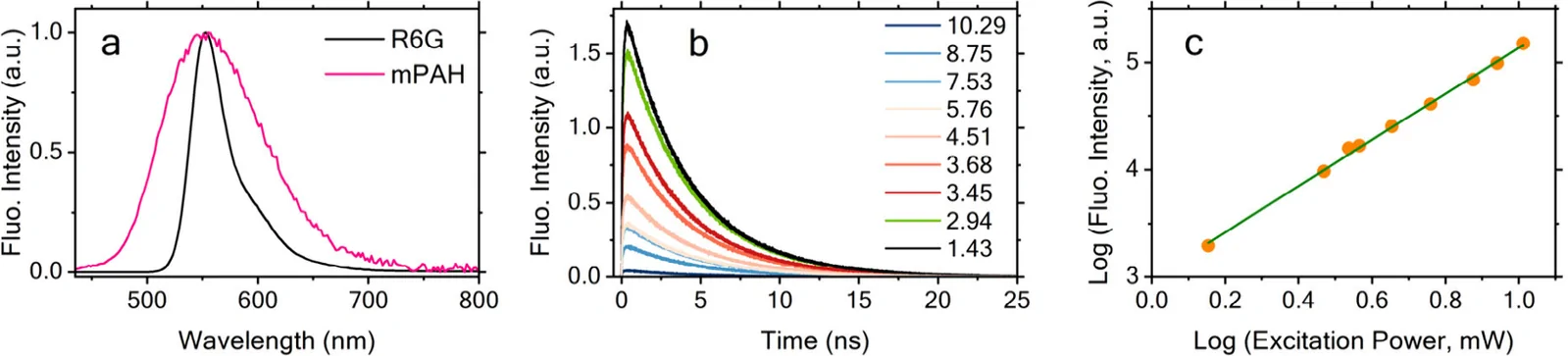
(a) Normalized steady-state fluorescence emission spectra acquired from dilute solutions of R6G in methanol and indazole mPAH in neat water upon excitation at 350 nm. (b) TRF decays measured using an 8 × 10^–6^ M methanol solution of R6G under different excitation intensities depicted by the corresponding average powers in mW. (c) Log–log plot of the time-integrated TRF intensities as a function of the average laser powers of the two-photon excitation. The solid line in (c) is a linear fit to the experimental data.

Observation of the TPEF emission from this mPAH first offers an opportunity to estimate its two-photon absorption cross-section, σ_*x*_, using a comparative method.^24,25^ By choosing a standard with well-defined cross-section σ_st_, σ_*x*_ can be expressed as

1 $$\sigma_{x}=\frac{10^{b_{x}-b_{\mathrm{st}}}\varphi_{\mathrm{st}}\sigma_{\mathrm{st}}{\left[c\right]}_{\mathrm{st}}n_{\mathrm{st}}}{\varphi_{x}{\left[c\right]}_{x}n_{x}}$$

where φ refers to the fluorescence quantum yield, *n* denotes the solvent refractive index, [*c*] stands for the concentration, *b* represents the intercept determined from the linear fit of the log–log plot of the TPEF intensity as a function of excitation power, and subscripts *x* and st refer to sample and standard, respectively.

We choose rhodamine 6G (R6G) as our standard based on its known σ_st_ in the literature^26^ in combination with its similar steady-state fluorescence emission spectrum as that observed for the mPAH as shown in Figure 2a. To determine the intercept *b*_st_, we perform TRF measurements under different excitation intensities on a methanol solution with an equal concentration as used for the reported cross-section quantification, *i.e.*, [*c*]_st_ = 8 × 10^–6^ M.^26^ The results, along with a plot of the time-integrated fluorescence intensity over the entire temporal range of the detectable emission as a function of the excitation power are shown in Figure 2b and 2c, respectively. A linear fit to the log–log plot shown in Figure 2c gives the intercept *b*_st_ = 2.99 ± 0.027 and a slope of 2.15 ± 0.038. In addition, both the fluorescence quantum yield of R6G methanol solution and methanol refractive index can be readily found in the literature, namely, φ_st_ = 0.93 ± 0.005 ^27^ and *n*_st_ = 1.3288 at 532 nm and 300 K.^28^

Similarly, several parameters are known for the mPAH sample too. Specifically, refractive index of water is available in the literature: *n*_*x*_ = 1.33538 at 532 nm and 20 °C,^29^ and the concentration chosen for the measurements was [*c*]_*x*_ = 1 × 10^–3^ *M*. Furthermore, the intercept *b*_*x*_ = 2.74 ± 0.05 can be found from the linear fit to the log–log plot shown in Figure 1c. This leaves the mPAH fluorescence quantum yield φ_*x*_ as the only unknown parameter in eq 1. We follow the procedure described in ref 30 to determine φ_*x*_, which can be written as follows,

2 $$\varphi_{x}=\varphi_{\mathrm{st}}\frac{F_{x}}{F_{\mathrm{st}}}\frac{f_{\mathrm{st}}}{f_{x}}\frac{n_{x}^{2}\left(\lambda_{\mathrm{em}}\right)}{n_{\mathrm{st}}^{2}\left(\lambda_{\mathrm{em}}\right)}$$

where *F* is the florescence intensity integrated over the entire emission spectral range, and *f* is the absorption factor, which can be written as *f* = 1 – 10^–*A*(λ_ex_)^ with *A* being the absorbance at the excitation wavelength *λ*_ex_. Based on the absorption spectra of the mPAH and R6G solutions, we obtained *f*_st_ = 0.02725 and *f*_*x*_ = 0.07743. The *F*_st_ and *F*_*x*_ values are obtained by integrating the fluorescence emission spectra over the entire spectral range from 435 to 800 nm, resulting *F*_st_ = 3.59452 × 10^8^ and *F*_*x*_ = 5.5038 × 10^6^. Based on these results, we calculated φ_x_ = 0.0051, the fluorescence quantum yield for this highly water-soluble indazole mPAH.

With all these parameters needed for quantifying σ_*x*_, we can readily obtain σ_x_ = 53.5 GM for this indazole mPAH. Although this two-photon excitation can initiate the photoreaction of this mPAH and in turn leads to the formation of other structurally distinct isomers such as *cis* and *spiro* etc.,^6,7,11,17,18^ we assign this cross-section exclusively to the *trans* isomer considering the fact that this estimate is made based on the fluorescence emission of the *trans* isomer. We note that this value is close to the σ_st_ = 65 ± 9 GM reported for a R6G methanol solution in a comparable spectral range (798–806 nm).^26^ This explains why TPEF emission can be readily observed from this mPAH, albeit their fluorescence quantum yields differ by over 180 times. This value is also only about 30% smaller than the σ = 80 GM determined for a chloroform solution of merocyanine at 700 nm using *Z*-scan technique.^20^ While aggregate formation may contribute to this reported value due to the use of concentrated solution (∼0.2% by weight), observation of successful photoreaction initiation for a merocyanine-based photoacid^21^ does indicate a sizeable σ for this specific photoacid.

While Tsutskiridze *et al*. have shown that the photoreaction of a merocyanine-based photoacid can be initiated by two-photon excitation with a lower efficiency than its one-photon excitation,^21^ it remains unexplored if the underlying reaction pathway resembles or differs from the one induced by one-photon excitation. Measurements of the TPEF decays and time-integrated emission spectrum enable us to gain insight into this unanswered question. First, we note that the two-photon excitation leads to a distinct TPEF decay from the one acquired using one-photon excitation. As shown in Figure 3, the TPEF decay at 560 nm exhibits a noticeably slower decay than that measured upon one-photon excitation. Quantitative analysis of these decay profiles was performed employing a least-squares deconvolution fitting algorithm with explicit consideration of the finite instrument response function (IRF) in combination with a model function consisting of a sum of exponential components. Using an IRF with a full width at half maximum (FWHM) of 48 ps determined using a pyridinium styryl dye, 2-[2-(4-dimethylamino-phenyl)-vinyl]-1-methylpyridinium iodide (o-DASPMI) in 10 mM phosphate buffered saline (pH 7.4) at the same emission wavelength,^31^ we found that a model function consisting of two exponential components is needed for satisfactory fit of the one-photon decay with the resulting lifetimes (relative amplitudes) of 25 ps (50.3%) and 60 ps (49.7%), respectively. In contrast, a model function consisting of three exponential components is necessary to describe satisfactorily the TPEF decay with the resolved lifetimes (relative amplitudes) of 23 ps (42.9%), 69 ps (56.0%), and 409 ps (1.2%), respectively. Although the relative amplitude of this slowest decay component is very small, its inclusion in the fitting is necessary to describe the long-lived fluorescence signal that is observed only with two-photon excitation. We note that this small relative amplitude corresponds to a much greater fractional intensity, which is given by $f_{i}=\frac{A_{i}\tau_{i}}{\underset{i}{\sum }A_{i}\tau_{i}}$ ­(*i* = 1, 2, 3), where the *A*_*i*_ and *τ*_*i*_ are the relative amplitude and lifetime of decay component *i*. For the three decay components determined for the TPEF decay (Figure 3), the corresponding fractional intensities are 18.5%, 72.3%, and 9.2%, respectively. This large fractional intensity further makes impurity in the sample an unlikely origin of the observed slowest decay component and the red-shifted fluorescence spectral component as shown below.

**Figure 3:**
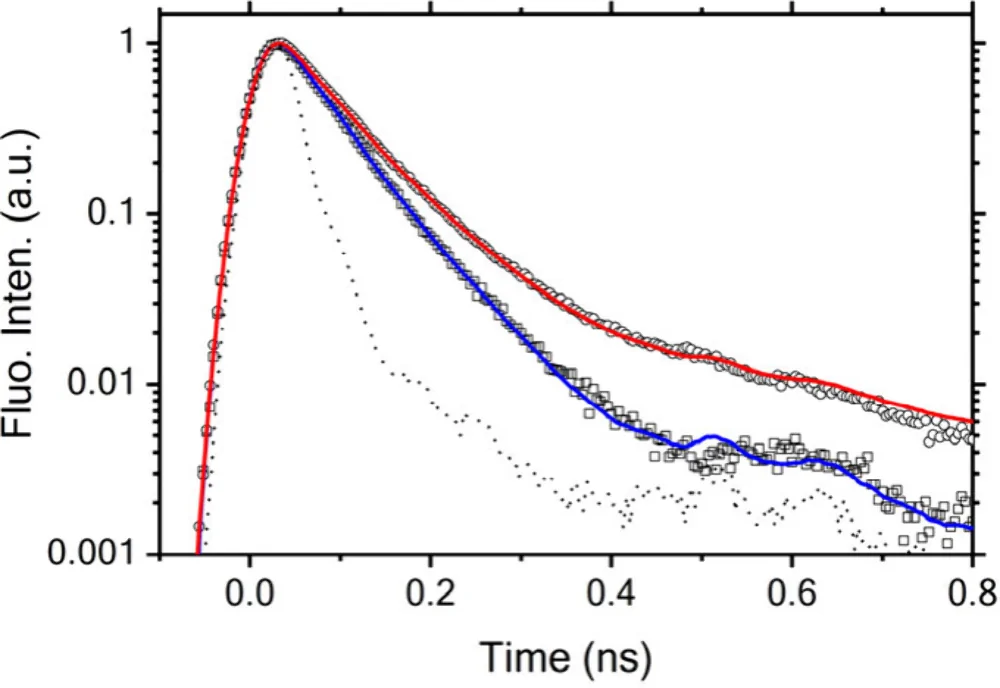
Normalized TRF decays measured at 560 nm upon one- and two-photon excitation (open squares and circles), respectively. The blue and red lines depict the deconvolution fits using the IRF shown by the short dash line.

Second, we further note that the time-integrated TPEF emission spectrum is different from the steady-state fluorescence emission spectrum acquired with one-photon excitation. To obtain such a time-integrated TPEF emission spectrum, we performed TFRF measurements over a spectral range from 450 to 750 nm and the resulting spectra are plotted as a function of the emission wavelength and time in Figure 4a. To calculate the time-integrated emission spectrum from the data shown in Figure 4a, we integrated the fluorescence intensities at each emission wavelength over the entire temporal range of the detectable emission, *i.e.*, along the vertical axis. As shown in Figure 4b, while the resulting time-integrated spectrum exhibits considerable similarity to the corresponding one-photon excited steady-state spectrum, a clear red shift and a distinct feature on the longer wavelength side are readily observable in the TPEF spectrum. Although the finite overlap between the absorption and TPEF emission spectra shown in Figure 4b may contribute partially to the observed spectral shift owing to the use of a high sample concentration for the TFRF measurement, the observed shift extends clearly beyond this overlap and thus cannot be caused by reabsorption. To assess the distinct feature on the longer wavelength side, we performed spectral deconvolution analysis for the TPEF spectrum. As shown in Figure 4c, two Gaussian components with peak wavelengths at 564.4 and 637.1 nm in combination with their corresponding FWHM of 71.8 and 75.5 nm are necessary to satisfactorily describe the TPEF spectrum. The observed distinct TPEF decay and time-integrated emission spectrum strongly suggest that different isomers are involved in one- and two-photon excitation. Existence of these isomers including those transient species has been reported by several groups.^2,11,17–19,22^ These isomers may be structurally similar but differ in their polarizability or hyperpolarizability, leading to distinct behavior for one- and two-photon excitation.^32,33^ Since the donor and acceptor parts of these isomers are the same, the difference in polarizability is directly related to their conjugation. Specifically, an isomer with the least conjugation and polarizability should possess moderate one-photon fluorescence but no or very weak two-photon fluorescence. Conversely, those with the largest conjugation and polarizability should possess high two-photon fluorescence but none or very weak one-photon fluorescence. This observation further indicates activation of different photoreaction pathways upon one- or two-photon excitation. Given the complicated reaction pathways of such mPAHs and associated electronic structures of various isomers,^2,17,18,23^ any attempt to identify the possible pathways induced by two-photon excitation will necessitate detailed theoretical calculations, which are beyond the scope of this letter.

**Figure 4:**
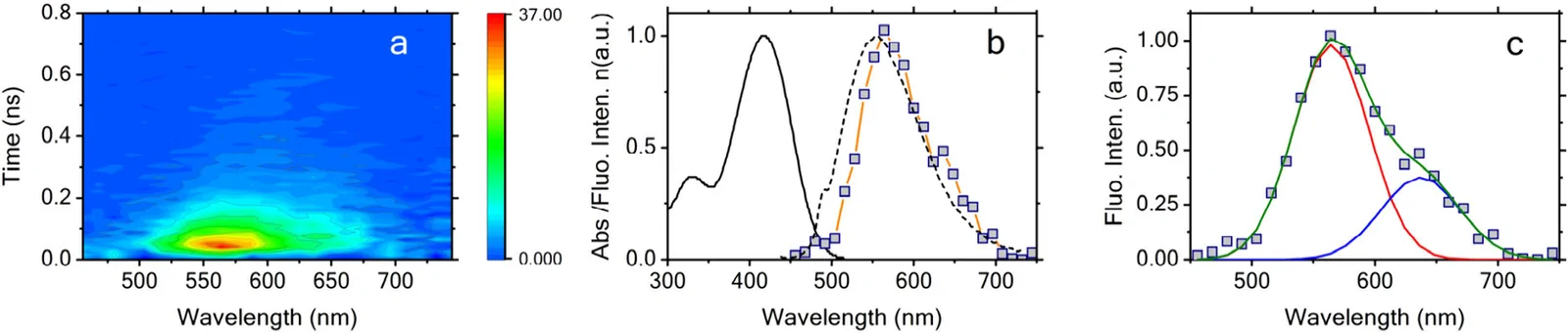
(a) Contour plot of the TFRF emission spectra acquired for a 1 mM aqueous mPAH solution upon two-photon excitation at 804 nm as a function of time (ns) and emission wavelength (nm). (b) Linear absorption (black line), steady-state fluorescence emission spectrum acquired upon excitation at 418 nm (short dash line), and time-integrated emission spectrum obtained by integrating TRF data over the entire time window with detectable signals shown in (a) (symbols and orange line). The spectra are scaled to an equal intensity at the peak wavelengths to ease comparison. (c) Spectral deconvolution analysis of TPEF emission spectrum (symbols), along with two distinct spectral components (blue and red lines) and the resulting overall fit (dark green line).

Two-photon excitation occurs either within a finite volume for a focused laser beam or along the laser propagation path for a non-focused high intensity laser beam. Consequently, the photoreaction and the resulting proton release is spatially confined, making its detection challenging for both pH and absorption spectral measurements. As detailed in the Supporting Information, upon irradiating with a non-focused beam with 1/*e* ^2^ diameter of 936 μm (see Figure S3) and an average power of 220 mW, which was kept below the threshold of white-light continuum generation, our pH measurements on aqueous solutions with a total volume of 8-10 ml using a conventional pH meter (Orion Star A211 with 8107BNUMD probe) lead to a pH change too small to be reliably validated. This is in stark contrast to the pH change from 6.55 to 3.88 upon irradiating the sample with a 470 nm LED for 5 min.^11^ This unsuccessful attempt is consistent with the estimated low proton concentration as detailed in the Supporting Information. While continuous irradiation of a sample solution over 7 h leads to a noticeable absorbance decrease of the *trans* isomer absorption peaking at 418 nm, we observed no absorption recovery upon keeping the sample solution in darkness for 1 h (see Figure S4). This observation does not mean that the two-photon initiated photoreaction is irreversible, but instead the resulting changes are too small for ensemble spectroscopic measurements. Future verification of this reversibility may be achieved through a microscopic approach capable of probing selectively the irradiation volume of two-photon excitation or, alternatively, irradiation with a high intensity, continuous wave near-infrared light source will create a sufficiently large effect for spectroscopic detection at an ensemble level.

In this study, we report, to the best of our knowledge, the first observation of TPEF emission from an aqueous solution of a highly water-soluble indazole mPAH. The readily observable fluorescence emission was found to not only follow the anticipated quadratic intensity dependence but also possess a sizeable two-photon absorption cross-section of ∼53 GM. This value is only about 30% smaller than the 80 GM determined for a merocyanine in chloroform at 700 nm using *Z*-scan technique.^20^ We further found that the TRF decay and time-integrated emission spectrum observed upon two-photon excitation differ clearly from the corresponding results obtained with one-photon excitation. This difference serves as indirect evidence for the activation of different isomers and their associated pathways in the photoreactions upon one- and two-photon excitation. Our findings pave a promising way of unlocking new functionalities and applications of these mPAHs that are inaccessible to one-photon excitation. Specifically, it can promote the formation of two-photon absorbing isomers with large conjugation and polarizability, which may offer new functionalities inaccessible for one-photon excitation. Direct two-photon excitation with enhanced penetration depth and tight focal volume will enable precise and targeted drug delivery and activation without the need of generating visible light for one-photon excitation using upconversion nanoparticles.^34,35^ It will further offer better temporal and spatial control of multiple mPAH-mediated processes such as activation of ion channels,^36^ ion sensing,^37^ dissipative assemblies,^4,38,39^ and transformations in synthesis.^21^ Moreover, such two-photon excitation will provide significant technical convenience to probe and unambiguously identify the different isomers involved in the complicated photoreaction of such a mPAH using ultrafast optical spectroscopic techniques. Since all irradiated mPAH molecules will be excited upon one-photon excitation, the slower reverse reaction rate than the repetition rates of commonly used pulsed laser systems means that repeated excitation of same or similar sample volume will unavoidably lead to accumulation of different isomers. Note that none of those commonly used approaches (rapid sample flow through excitation volume or use of fast-spinning sample cell) is fast enough to refresh the sample for each successive laser pulse. Consequently, identification of the spectral and dynamics associated with each isomer is challenging, if not impossible. In contrast, the well-defined excitation volume afforded by two-photon excitation using focused laser beam should make access to a fresh sample volume possible for each successive laser pulse and thus may overcome this technical challenge.

## Experimental Methods

A. *Materials*. Synthesis, purification and characterization of the water-soluble indazole mPAH with a solubility of about 30 mM was performed following the procedure described previously.^11^ An aqueous solution of 1 mM was prepared for all TPEF measurements using ultrapure water (18.2 MΩ·cm). Upon two-photon excitation with both focused and non-focused laser beams of various average powers ranging from ∼1 mW to 220 mW, our measurements on this and other samples with comparable concentrations over continuous irradiation from 7 to 16 h show the largest absorbance decrease of ∼5% at 418 nm, the peak wavelength of *trans* isomer absorption. This observation is strikingly distinct from the substantial changes observed for one-photon excitation of a different indazole mPAH over a much short exposure time (>1 h),^17^ and thus it can serve as direct evidence for an exceptional chemical stability of the indazole mPAH under two-photon excitation and the resulting confined excitation volume. An example of the absorption spectra acquired after 7 h continuous irradiation to a non-focused, high intensity laser beam with an average power of 220 mW is shown in Figure S4.

Rhodamine 6G (R6G, Exciton) was chosen as the standard for two-photon absorption cross section (σ_st_) determination. A methanol solution with a concentration of 8 × 10^–6^ M as used for measuring the reported σ_st_ value was prepared using spectroscopic grade methanol. Accurate concentration adjustment was made by setting its absorbance at 530 nm to 0.928 per cm based on the reported molar extinction coefficient of 116000 cm^–1^ M^–1^ for R6G in methanol (529.8 nm).^40^

B. *Absorption Measurements*. A Shimadzu UV-1900i spectrometer was used to measure all absorption spectra reported in this work.

C. *Steady-State Fluorescence Measurements*. Relative fluorescence quantum yield of the mPAH was determined following the procedure described in ref. 30 using 6G as the standard. A Fluorolog-QM spectrofluorometer equipped with single grating excitation and emission monochromators was used to record steady-state fluorescence spectra of the mPAH sample and the R6G standard. Corrected fluorescence emission spectra were collected from 435 to 800 nm upon excitation at 350 nm using a 2 nm bandwidth for both excitation and emission monochromators, a step size of 2 nm and an integration time of 1 s. A dilute aqueous mPAH solution with a maximum absorbance of 0.073 in 1 cm × 1 cm quartz cuvette was prepared using a fresh stock solution to avoid occurrence of its photoreaction prior to measurement. Similarly, a methanol R6G solution in a same size cuvette was also diluted to a maximum absorbance of 0.085.

D. *Time-Resolved Fluorescence Measurements*. TFRF measurements upon one- and two-photon excitation were performed using a time-correlated single-photon-counting (TCSPC) apparatus (PicoHarp 300, Picoquant) as described previously.^18^ Briefly, the excitation light was a small portion of the output of a 250 kHz Ti:sapphire femtosecond regenerative amplifier (RegA 9050, Coherent) capable of generating typically 50 fs, 6 μJ pulses at 804 nm. For one-photon excitation measurements, the 804 nm pulses were further frequency doubled using a 2 mm thick BBO crystal to obtain excitation pulses centered at 402 nm. The detection combines an ultra-stable Gemini Interferometer (Nireos SRL) for frequency-resolved detection capability, and a hybrid photomultiplier detector assembly (PMA Hybrid 07, PicoQuant) with a timing resolution <50 ps (FWHM).^18^ Measurements of the intensity dependence of both mPAH and R6G samples were performed by acquiring TRF data at a fixed emission wavelength of 560 nm using a bandpass filter with a FWHM of 10 nm (FBH560-10, Thorlabs) under different excitation intensities. A short pass filter (770/SP, Semrock) was also used to ensure complete elimination of the pump light scattering from reaching the detector. During these measurements, the Gemini Interferometer was set at a fixed, zero-time delay. A 4.0 ps channel time and a fixed integration time were chosen for all TRF data acquisition. To determine the fluorescence intensity at a given excitation intensity, we integrated the TRF data over the entire time range of the detectable signal.

Acquisition of TFRF data on the aqueous mPAH solution upon two-photon excitation was performed following a similar procedure as described previously for one-photon excitation. To detect the emission over the entire spectral range, we replaced the 560 nm bandpass filter with a second short pass filter (FESH0750, Thorlabs). A laser power of 4.5 mW was chosen for the two-photon excitation.

## Supplementary Material



## Notes

Determination of the reverse reaction rate, generation of macroscopically visible two-photon excited fluorescence (TPEF), estimate of two-photon excited proton concentration, absorption spectra upon two-photon excitation, supporting experimental results including Figures S1–S4, and supporting references (PDF)
