# Supplementary material for: Activating Photoreaction of Indazole Metastable-State Photoacid through Two-Photon Excitation
Source: J Phys Chem Lett. 2026 Sep 12;17(38):10839–45. doi: 10.1021/acs.jpclett.6c02370 (PMC13618034; doi:10.1021/acs.jpclett.6c02370)
Supplement: Supplementary file 1 [file jz6c02370_si_001.pdf]

Supporting Information for

Activating Photoreaction of Indazole Metastable-State Photoacid through Two-photon Excitation

Ying-Zhong Ma[a*], Pavithra Liyanage[b], and Yi Liao[b]

[a]Chemical Sciences Division, Oak Ridge National Laboratory, Oak Ridge, Tennessee 37831

[b]Department of Chemistry and Chemical Engineering, Florida Institute of Technology, Melbourne, Florida 32901

*Corresponding author: may1@ornl.gov

Notice: This manuscript has been authored by UT-Battelle, LLC under Contract No. DE-AC05-00OR22725 with the U.S. Department of Energy. The United States Government retains and the publisher, by accepting the article for publication, acknowledges that the United States Government retains a non-exclusive, paid-up, irrevocable, world-wide license to publish or reproduce the published form of this manuscript, or allow others to do so, for United States Government purposes. The Department of Energy will provide public access to these results of federally sponsored research in accordance with the DOE Public Access Plan (http://energy.gov/downloads/doe-public-access-plan).

Determination of the reverse reaction rate

We measured the reverse reaction rate by acquiring absorption spectra at different times after irradiation with a 400 nm LED array. As shown in Figure S1 (left panel), the absorption spectra recover in 20 min. By plotting the absorbance at 416 nm vs. time and fitting the resulting data into a first order reaction equation as shown in Figure S1 (right panel), which is commonly used for mPAHs[1], we found that the reverse reaction rate is 1.6 x 10^{-3} s^{-1}, corresponding to a half-life of 433 s or 7.2 minutes.

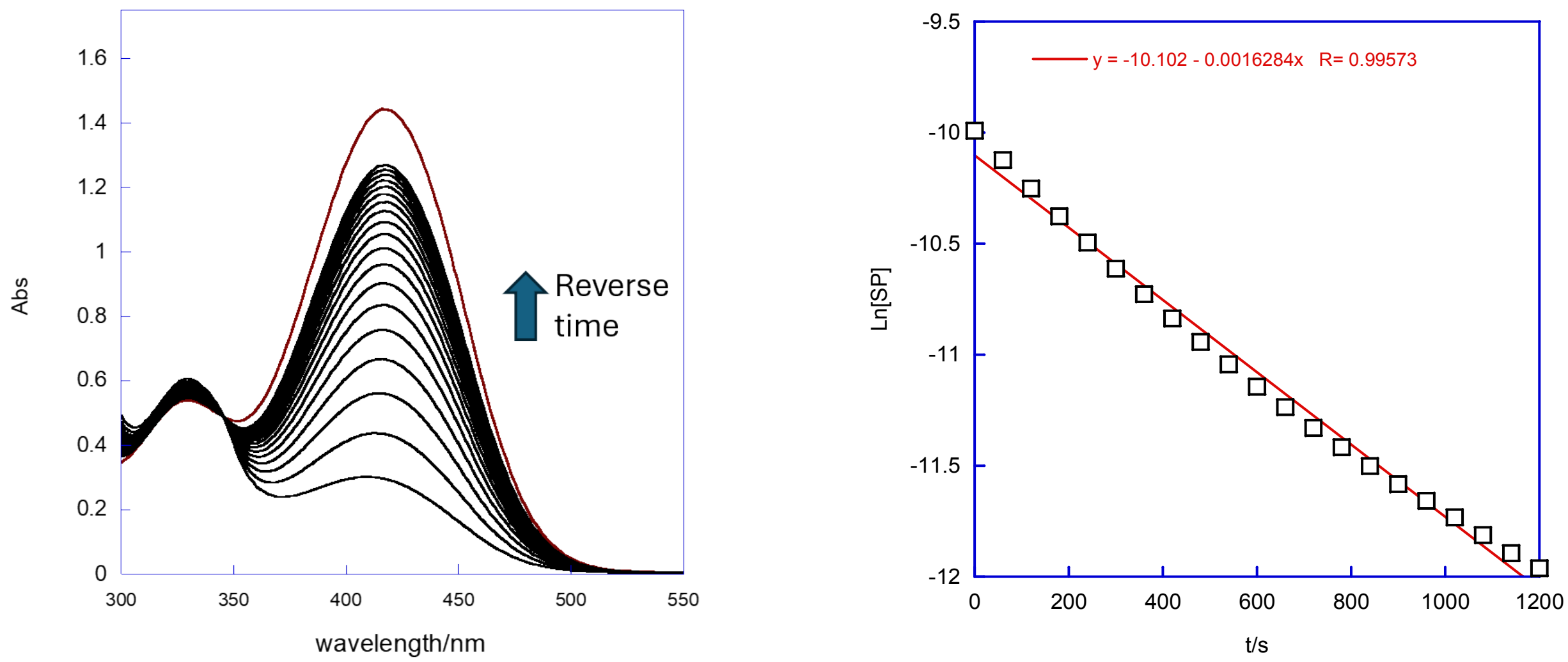

Figure S1. (Left) Absorption spectra of an aqueous solution of the indazole mPAH acquired at various times after irradiation. The red curve is the spectrum before irradiation. (Right) Plot of absorbance at 416 nm vs. time and its fitting to a first order equation.

Generation of macroscopically visible two-photon excited fluorescence (TPEF)

Irradiation of a 1 cm fluorescence cuvette filled with a 0.53 mM aqueous solution of indazole mPAH using a non-focused, high intensity femtosecond laser beam with an average power of 220 mW or a loosely focused, intermediate intensity beam of 23.2 mW leads to TPEF generation along the excitation propagation direction, which is sufficiently intense to be visible for a naked eye (See Figure S2). Note that this is different from the TPEF generation using focused laser beam, where the emission is expected to confine in a small focal volume.

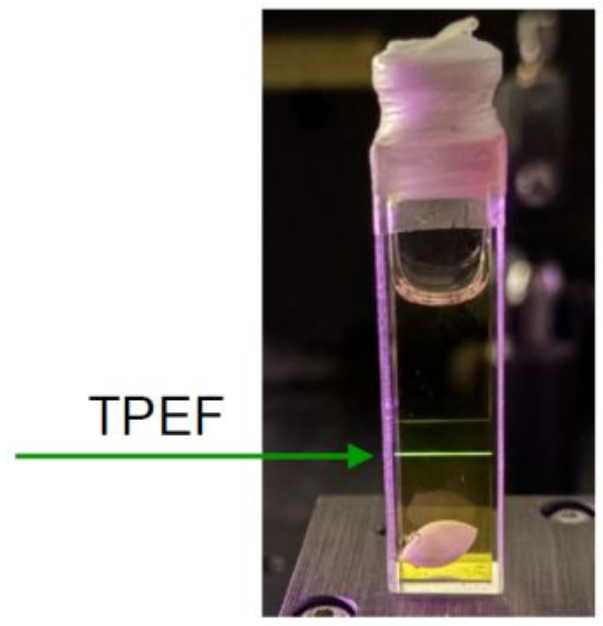

Figure S2. A photograph of the airtight cuvette containing aqueous solution of 0.53 mM indazole mPAH under irradiation of a loosely focused, intermediate intensity femtosecond laser beam with an average power of 23.2 mW. The horizontal yellow line indicated by the arrow is the TPEF.

Estimate of two-photon excited proton concentration

This estimate was made following the procedure described by Liao.[2] Specifically, when the reverse reaction is a first order reaction with a rate constant of $k = ln2/\tau$ with τ being the half-life of the acid state, the proton concentration can be expressed as

$$[H^+] = qf\phi\tau/(\ln 2 \cdot L), \quad (1)$$

where q is photon flux, f is the fraction of the photon absorbed in the two-photon excitation process, ϕ is the quantum yield, and L is the path length of excitation light in the sample. To generate sufficiently high proton concentration for a reliable estimate, we employed an average laser power of 220 mW, which is approximately 20 times higher than the highest power used for the experiments described in the manuscript. This laser beam was used without focusing to avoid the generation of white-light continuum from the cuvette windows and the solvent (water). A 1 cm quartz cuvette was positioned precisely to ensure that (1) its reflection overlaps with the incident beam, and (2) the same cuvette window faces the incident laser beam for measuring the laser power behind the cuvette filled with neat water and the mPAH solution, respectively. A fresh aqueous solution of 0.53 mM indazole mPAH was gently stirred using an electronic cell stirrer (Starna Spinette) during the entire irradiation time.

To determine the photon flux q, both the beam spot size and the laser power attenuation by the sample need to be measured. As shown in Figure S3, the spot size has a $1/e^2$ diameter of 936 μm based on the measurement using a knife edge approach. To quantify the portion of excitation light being absorbed by the mPAH solution, we performed measurements on both neat water and the aqueous mPAH solution using a Thorlabs power meter (PM100D with S302C thermal power sensor head) positioned right behind the cuvette, leading to a difference of 1.3 mW. Based on these values, we can readily obtain $q = 5.04 \times 10^{-9}$ mmol·cm^{-2}·pulse^{-1}.

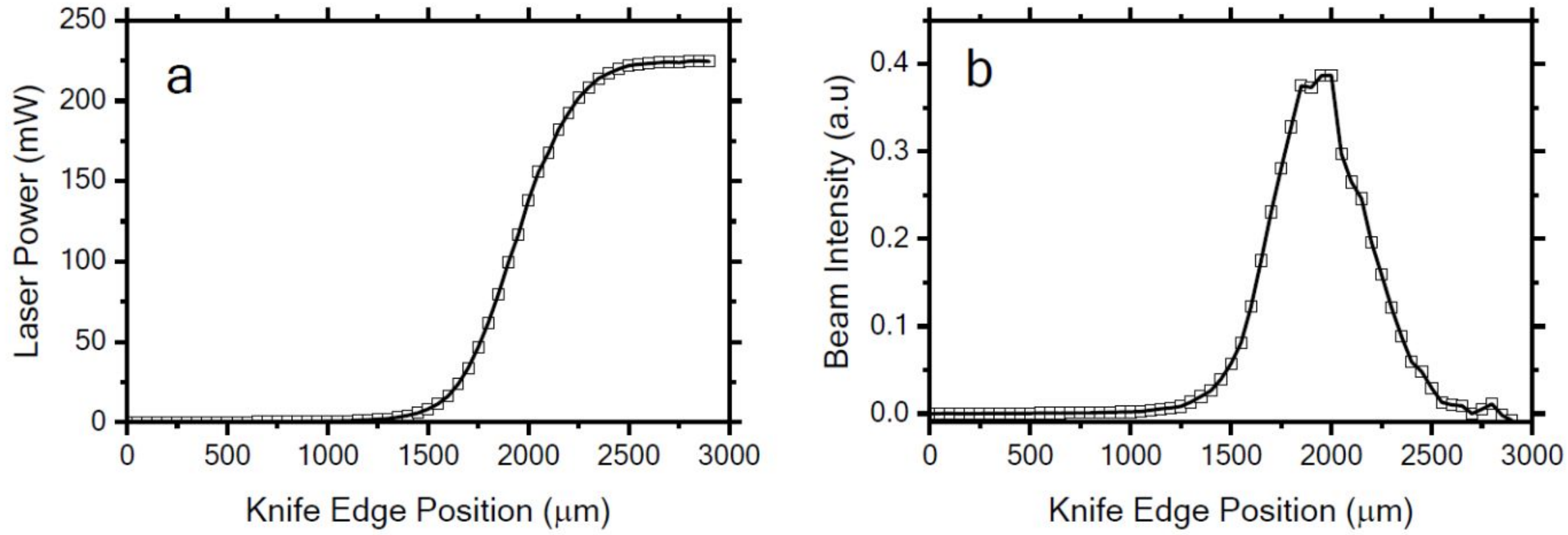

Figure S3. (a) Two-photon excitation laser power as a function of a knife edge position, and (b) the first derivate of the data in (a) showing a $1/e^2$ diameter of 936 μm.

The quantum yield of two-photon excitation can be calculated using the following equation:

$$\phi = \frac{1}{2}\sigma g F_0^2 \tau, \tag{2}$$

where σ is two-photon absorption cross-section, g is the second-order temporal coherence factor, F_0 is the peak photon flux density, and τ is the pulse width. For $F_0 = 1.217 \times 10^{26}$ photons·cm^{-2}·s^{-1}, $\sigma = 53$ GM, $g \approx 0.664$ for Gaussian pulses, and $\tau = 50$ fs, we find $\phi \approx 1.30 \times 10^{-10}$. Since q is calculated based on the measured fraction of the photon absorbed in the two-photon excitation process, we can safely set $f = 1$. With these values and conversion from pulse^{-1} to s^{-1} by multiplying the laser repetition rate, we obtain $[H^+] = 1.023 \times 10^{-10}$ M for the proton concentration in the sample volume irradiated by this non-focused laser beam. This low proton concentration explains why our bulk pH measurements on aqueous solutions with a total volume of 8-10 ml using a conventional pH meter (Orion Star A211 with 8107BNUMD probe) were unsuccessful. Note that upon focusing the laser beam, the proton concentration should be greater within the focal volume.

Absorption spectra upon two-photon excitation

To examine if two-photon excitation can induce a same or similar photoreaction as commonly observed for one-photon irradiation, we measured absorption spectra by withdrawing small volumes from the 1 cm cuvette containing an aqueous solution of 0.53 mM indazole mPAH before and after 7 hours irradiation using a non-focused, high intensity femtosecond laser beam with an average power of 220 mW. As shown in Figure S4, the irradiation leads to a noticeable absorbance decrease of the *trans* isomer absorption peaking at 418 nm, whereas the corresponding absorption change of the *cis* isomer around 330 nm appears much smaller. Upon keeping the sample solution in darkness for 1 hour, the absorption spectrum exhibits a negligible change instead of the anticipated absorption recovery. Nevertheless, this observed decrease of the *trans* isomer absorption indicates that the two-photon excitation of this indazole mPAH may be able to undergo a similar photoreaction as found for one-photon excitation, albeit it is much less effective in

altering the bulk solution properties that can be detected by ensemble spectroscopic measurements. This low efficiency of two-photon excitation with respect to the one-photon visible light absorption process was observed previously by Tsutskiridze *et al.* for the photoreaction of a merocyanine-based photoacid.[3] Obviously, a microscopic approach capable of probing selectively the irradiation volume of two-photon excitation would be highly desirable. Alternatively, irradiation with a high intensity, continuous wave near-infrared light source will create more significant effects at an ensemble level.

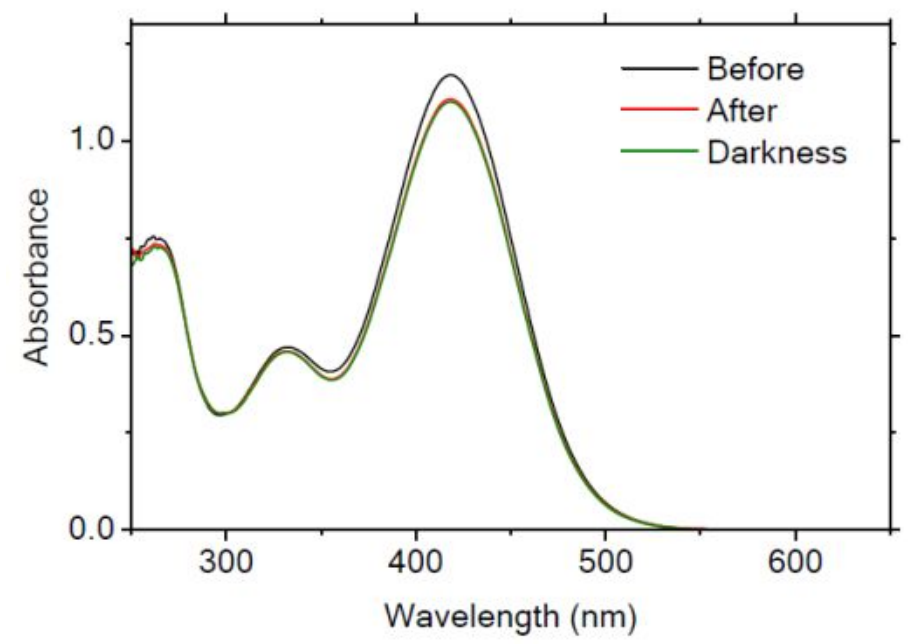

Figure S4. Absorption spectra acquired before (black line) and after 7 hours irradiation with non-focused, high intensity femtosecond laser beam with an average power of 220 mW (rad line). The spectrum measured after further keeping the sample in darkness for 1 hour (olive line) exhibits no difference from the radiated one.

Reference:

(1) Abbood, R.; Khan, S. N.; Liyanage, P.; Wang, Z.; Bykov, D.; Bryantsev, V. S.; Liao, Y. Metastable-State Photoacids with Fast Reverse Reactions. *ChemPhotoChem* **2026**, *10* (1), e202500223. DOI: 10.1002/cptc.202500223.

(2) Liao, Y. Reversible photo control of proton chemistry. *Phys. Chem. Chem. Phys.* **2022**, *24*, 4116-4124. DOI: 10.1039/d1cp05627a.

(3) Tsutskiridze, A.; Králová, D.; Ghosh, I.; König, B. Merocyanine-based photoacids as recyclable catalysts in visible-light-driven transformations. *Chem. Commun.* **2025**, *61* (36), 6651-6654. DOI: 10.1039/D5CC00696A.
